# The pVHL neglected functions, a tale of hypoxia-dependent and -independent regulations in cancer

**DOI:** 10.1098/rsob.200109

**Published:** 2020-07-01

**Authors:** Giovanni Minervini, Maria Pennuto, Silvio C. E. Tosatto

**Affiliations:** 1 Department of Biomedical Sciences, University of Padova, Viale G. Colombo 3, 35121 Padova, Italy; 2 Veneto Institute of Molecular Medicine, Via Orus 2, 35129 Padova, Italy

**Keywords:** VHL disease, hypoxia response, protein degradation, angiogenesis, cancerogenesis, tumour suppressor

## Abstract

The von Hippel–Lindau protein (pVHL) is a tumour suppressor mainly known for its role as master regulator of hypoxia-inducible factor (HIF) activity. Functional inactivation of pVHL is causative of the von Hippel–Lindau disease, an inherited predisposition to develop different cancers. Due to its impact on human health, pVHL has been widely studied in the last few decades. However, investigations mostly focus on its role in degrading HIFs, whereas alternative pVHL protein–protein interactions and functions are insistently surfacing in the literature. In this review, we analyse these almost neglected functions by dissecting specific conditions in which pVHL is proposed to have differential roles in promoting cancer. We reviewed its role in regulating phosphorylation as a number of works suggest pVHL to act as an inhibitor by either degrading or promoting downregulation of specific kinases. Further, we summarize hypoxia-dependent and -independent pVHL interactions with multiple protein partners and discuss their implications in tumorigenesis.

## 1. Introduction

Functional inactivation of the von Hippel–Lindau protein (pVHL) is causative of the so-called von Hippel–Lindau (VHL) disease, a familiar predisposition to develop cancer [1–3]. VHL disease is characterized by the progressive development of multiple tumours affecting specific target organs, such as the retina, adrenal glands, epididymis, pancreas and kidneys [1,4,5]. It is a severe autosomal dominant genetic condition with inheritance of one in over 35 000 [6,7]. The homonymous *VHL* gene codifying for pVHL protein localizes on chromosome 3p25 and is constitutively expressed in both fetal and adult tissues [8]. It contains three exons, namely E1, E2 and E3 that encode a full length 213 amino acids protein pVHL30 [4] (also referred as pVHL213). A second internal translation initiation at the codon 54 methionine produces a shorter 160 amino acid protein named pVHL19 [9] (also known as pVHL160). A third 172 amino acids isoform, pVHL172, is generated by an alternatively spliced mRNA in which exon E2 is excluded [8,10]. To add complexity to the human *VHL* splicing regulation, further pVHL isoforms were recently proposed [11]. These putative isoforms are generated by inclusion of a novel E1′ cryptic exon of unknown function that contains an intronic sequence [11]. In particular, Lenglet *et al.* propose a promising novel 193 amino acids X1 protein isoform which seems to be conserved only in higher primates [11]. Both the two major gene products, pVHL30 and pVHL19, act as tumour suppressor and form an ubiquitin E3 ligase complex known as VCB [12,13] binding elongins B and C and cullin 2, whereas pVHL172 is thought to contribute to renal carcinoma development by upregulating a subset of pro-tumorigenic genes [14]. The main difference between the two main isoforms is that pVHL19 lacks the first 53 residues which form a mainly acidic and intrinsically disordered N-terminal tail [15]. The best known pVHL function is the ubiquitin-mediated degradation of hypoxia-inducible factor 1-alpha (HIF-1*α*) [3] under physiological oxygen concentrations. The interaction between pVHL and HIF-1*α* requires the prolyl-4 hydroxylase domain enzymes (PHD1, -2 and -3)-dependent hydroxylation [16] of at least one of two specific proline residues of HIF-1*α* (i.e. Pro402 and Pro564) localized within a conserved LxxLAP consensus sequence [17]. This motif was initially believed to be highly specific for PHD activity, while recent findings show that PHD can hydroxylate other prolines in a LxxLAP-independent fashion [18–22]. Hypoxic conditions inhibit PHD activity allowing HIF-1*α* to escape proteolysis and translocate to the nucleus, where it promotes transcription of many genes involved in angiogenesis, glucose metabolism, cell survival and tumour progression [3,23]. The pVHL is commonly described as a molecular hub, mediating interactions with more than 500 different proteins [24,25]. pVHL has negligible sequence identity to other human proteins, and although being well conserved among mammals [26], it shows important differences within this group. In particular, pVHL30 presents an intrinsically disordered [27] N-terminus containing multiple repetitions of an acidic pentamer in human and higher primates, whereas this same region is shorter and almost lacking repeated elements in rodents and lower mammals [15]. Kidney-specific pVHL inactivation causes the development of kidney cysts in a mouse model [28], while reintroduction of a wild-type gene interrupts malignant progression [29]. Nevertheless, pVHL somatic inactivation is calculated to affect about 75% of sporadic clear cell renal cell carcinomas (ccRCC) [30,31], while a number of studies suggests a role for pVHL in the regulation of the cellular tumour antigen p53 (p53) [32–34].

Several other HIF-1*α* independent functions are emerging in the literature [35–41]. In this review, we focus our efforts in describing these almost neglected pVHL functions.

### 1.1. von Hippel–Lindau protein and the regulation of kinases activity

**Figure 1.**
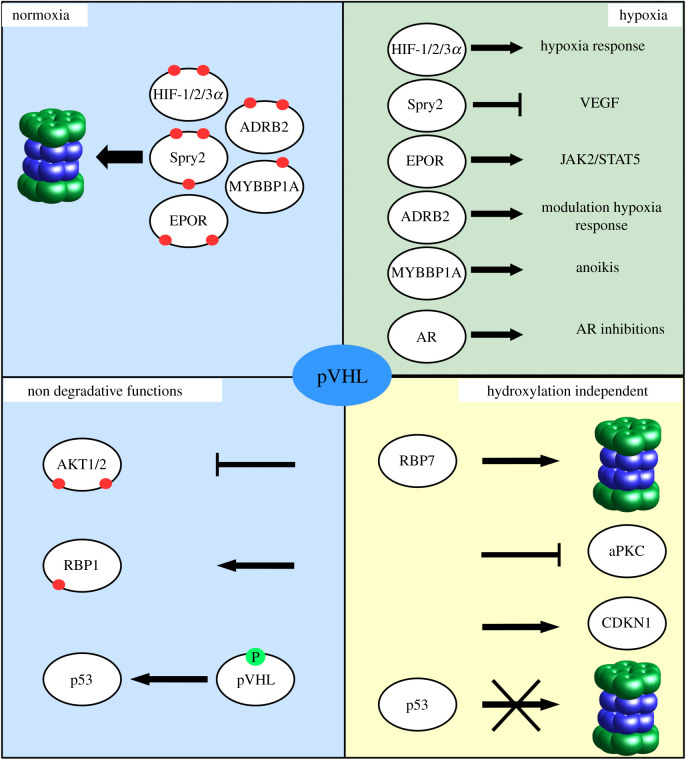
Overview of pVHL functions in four different scenarios. In normoxia, pVHL marks different proteins for proteosomal degradation by binding hydroxylated linear motifs (red dots). pVHL targets escape degradation during hypoxia activating a generalized cellular adaptation to oxygen deprivation. Hydroxylation-dependent interactions not finalized to protein degradation are also reported, such as AKT1/2 kinases inhibition. pVHL is itself regulated by phosphorylation (green dot) and is thought to exert a number of hydroxylation-independent functions.

Since its discovery pVHL was associated with hypoxia sensing and angiogenesis [42,43]. However, other functions in addition to the degradation of HIF-1/2*α* transcription factors were immediately proposed. In particular, early evidence suggested an alternative role for pVHL in the regulation of kinases activity (figure 1). In 1999, Shuin and co-workers [44] observed that the pVHL β-domain, which is the HIF-1/2*α* recognition module, interacts directly with atypical PKC isotypes, PKCz and PKC *λ*/l. The PKC*λ*/l belongs to the third group of the PKC family [45] and they play a relevant role in many cellular functions such as proliferation, differentiation and cell survival [45,46]. Shuin and co-workers also reported that the regulatory domain of PKC is sufficient for pVHL-PKCz and -PKC λ/l interactions. Further, their work demonstrated that the association between pVHL and aPKC occurs after aPKC activation suggesting that pVHL may directly impair or limit the aPKC function. Confirmation of this data was produced a few years later when the pVHL-dependent ubiquitination and degradation of aPKC was reported [47]. aPKC isoforms, in particular PKC*δ* and PKC*ζ*, have been shown to upregulate vascular endothelial growth factor (VEGF) expression, which is one of the HIF-1*α* main gene targets, by activating MAPK (mitogen-activated protein kinase) in pVHL defective cells [48]. More recently, the direct interaction between pVHL and PKC*δ* (protein kinase C *δ*) was also described [49]. Conversely to what was observed in aPKC, the interaction with PKC*δ* seems to not yield degradation but rather kinase inhibition. The interactions described so far are not mediated by proline hydroxylation, suggesting that they may occur irrespective of hypoxia conditions. An opposite scenario is proposed for the interaction between pVHL and AKT1 [21]. AKT1 is the isoform one of the AKT kinase family, which regulates many processes including metabolism, proliferation, cell survival, growth and angiogenesis. The interaction with pVHL requires the hydroxylation of specific proline residues of AKT1 [21] (table 1). AKT1 hydroxylation is mediated by PHD2, which in turn is the main regulator of HIF-1*α* hydroxylation [23,50–52]. The interaction promotes AKT1 functional inhibition, while, similarly to what was observed for PKC*δ*, it seems to have no effect on AKT1 degradation. Similar results are obtained for both AKT1 and AKT2 but not for AKT3. Intriguingly, the authors observed that the hydroxyl-prolines mediating these interactions reside within two FOXO-like linear motifs rather than the expected CODD-like motif. Further, the authors conducted site-directed mutagenesis of residues within the pVHL hydroxyl-proline binding pocket, showing that residues driving the binding to AKT1 and HIF-1*α* partially overlap but are not identical. These data, coupled with the difference in the linear motifs required for the interaction, strongly suggest that the pVHL β-domain may evolved to exert other functions over the sole HIF-1/2*α* recognition. Recently, NEK8 (never in mitosis gene A (NIMA)-related kinase 8) was reported to be downregulated by pVHL [53,54]. In particular, it was proposed to be a novel target of pVHL [54]. The best understood function of NEK8 is its role in cilia development in kidney cells [55], which is also one of the main HIF-1*α* independent pathways attributed to the pVHL function. NEK8 is also known to be an effector of the ATR-mediated replication stress response, a component of the DNA damage response linked to cystic kidney disorders [56]. pVHL is thought to either downregulate NEK8 through HIF-1*α* to maintain the primary cilia structure in human renal cancer cells [53] or to directly ubiquitinate NEK8 for proteosomal degradation [54]. These data show that functional interplays between kinases and pVHL can be achieved following different paths. Further, correlation between pVHL and kinase activity is not limited to the direct association with kinases. In a previous work, we reported the direct interaction between pVHL and the CDKN1 kinase inhibitor protein family [57]. We found that the CDKN1 proteins share a conserved region mimicking the HIF-1*α* motif responsible for pVHL binding. Of note, the binding does not require proline hydroxylation, while CDKN1b site-specific mutation associated with cancer is shown to modulate this novel interaction. The pVHL is also a substrate of different kinases [58–62] and the functional meaning of these modifications is only partially understood. Roe *et al.* [60] reported that the phosphorylation of pVHL Ser111 by Chk2 (checkpoint kinase 2) enhances pVHL-mediated transactivation of p53 on DNA damage by recruiting p300 and Tip60 to the chromatin of p53 target genes. pVHL is also known to bind p53 [63], this interaction promotes p53 stabilization through inhibition of MDM2-mediated degradation in a HIF-1*α* independent fashion. The observation that Chk2 regulates the pVHL-mediated transactivation of p53 suggests a novel rationale to understand how pVHL mutants may regulate cancer insurgence in VHL disease.

**Table 1.** Overview of pVHL interactors.

protein	hydroxylated residue	enzyme	degradation
hydroxylation dependent			
HIF-1*α*	P402, P564	PHD	Y
EPAS1	P405, P531	PHD	Y
HIF-3*α*	P492		Y
AKT1	P125, P313	PHD2	N
SPRY2	P18, P144, P160	PHD	Y
RBP1 (POLR2A)	P1465	PHD1	N
ADRB2	P382, P395	PHD3	Y
MYBBP1A	P695	PHD1	Y
EPOR	P443, P450	PHD3	Y
hydroxylation independent			
AR	N.A.	N.A.	N.A.
RBP7 (POLR2G)	N.A.	N.A.	Y
aPKC	N.A.	N.A.	N

### 1.2. von Hippel–Lindau protein targets for degradation RNA polymerase II subunits

The main function of pVHL as E3-ligase is to direct proteasomal degradation of HIF1/2-α under physiological oxygen conditions via the ubiquitin-proteasome pathway [64]. Two RNA polymerase II subunits, i.e. RBP1 and RBP7, are also reported to be marked for ubiquitination by pVHL [65,66]. Although both are essential components of RNA polymerase II complex, the interaction with pVHL is driven by two different mechanisms yielding different fates. Ubiquitination of RBP1 requires hydroxylation of a proline (i.e. Pro1465) within a CODD-like motif [67] partially resembling the pVHL/HIF-1*α* interaction [66]. Unexpectedly, the authors report [67] that RBP1 ubiquitination does not promote degradation but rather it correlates with intracellular accumulation of RBP1. Further, they observed that the ubiquitination of RBP1 is promoted by ultraviolet C (UVC) irradiation, which induces pVHL to associate with only active RBP1 that is engaged in transcription elongation. Coupling this finding with the pVHL-mediated transactivation of p53 on DNA damage [60] is easy to propose that these two mechanisms might cooperate for maintaining efficient DNA repair in response to DNA damaging factors. Different fate is for the pVHL/RBP7 interaction [65]. Indeed, this interaction is reported to be hydroxylation-independent and promote proteasomal degradation of RBP7. RBP7 is an essential protein [68,69] that plays a relevant role in starving cells that enter stationary phase [70]. The functional meaning of this association is not completely understood. Considering the independency from hydroxylation, it can be argued that association with pVHL is increased after hypoxic events and it may participate in the modulation of cell stress response.

### 1.3. von Hippel–Lindau protein associates with the androgen receptor

Sex is a key factor affecting the etiology, pathogenesis and prognosis of specific types of cancer. About 4.6% male and 2.7% female individuals develop kidney cancer, and the genetic basis for this sex discrepancy is unknown. The two- to fourfold higher risk of males to develop cancer compared with females is independent of geographical regions and socio-economic level, thereby implying that this sex disparity results from unknown genetic biological factors [71–73]. Rather, the higher incidence of renal cell carcinoma (RCC) in males compared with females suggests a role for sex hormones and their receptors in the onset, progression and outcome of disease. Androgen receptor (AR) and its natural ligands, testosterone and dihydrotestosterone, regulate the development of primary and secondary sexual characteristics during development. Mutations in the AR cause different types of androgen-related diseases depending on the mechanism involved. AR is expressed in about 15–20% of cancer tissues derived from RCC patients [74–78]. Importantly, sex hormone-based therapy showed effects in RCC patients that may suggest tumour endocrine dependence, yet not responsiveness [75,79–81]. Interestingly, the higher incidence of RCC in males has been shown to correlate with higher AR expression, suggesting the involvement of androgen signalling in the gender discrepancy of RCC [82,83]. Recently, the AR degradation-promoting compound, dimethylcurcumin (ASC-J9), has been shown to suppress RCC proliferation through a mechanism involving VEGF and HIF1*α*, providing experimental evidence for a role of AR and androgen signalling in RCC progression. pVHL has been shown to form a complex with AR in cultured cells [84,85]. However, the functional role of the pVHL/AR interaction remains unclear. pVHL has been shown to polyubiquitinate AR at lysines 845 and 847 and inhibit its transactivation [84]. However, it was not clear whether pVHL interaction with AR also regulates AR turnover. On the other hand, pVHL has been shown to enhance AR deubiquitination and reduce AR transactivation, suggesting non-canonical functions of pVHL in the regulation of AR function [85].

### 1.4. Multiple hydroxy-degrons regulate protein sprouty homologue 2, erythropoietin receptor, beta2-adrenergic receptor and Myb-binding protein 1A

**Figure 2.**
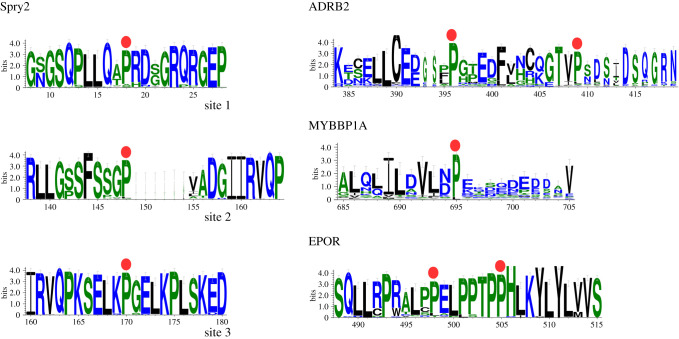
Consensus sequence of hydroxy-degrons driving the interaction between pVHL and its binding partners. Sequence comparison highlights that pVHL recognizes different apparently unrelated motifs. Red dots represent hydroxylation sites, while logo representations were generated aligning orthologous sequences retrieved from OMA browser [86], 10 residues upstream/downstream of the hydroxylation site were considered.

As E3-ligase component, pVHL seems to be specialized to bind short intrinsically disordered region containing a hydroxylated proline residue. The original linear motif described to be critical for pVHL/HIF-1*α* recognition was the LxxLAP motif within the so-called NODD and CODD fragments of HIF-1*α* [23]. The same motif was also believed to be highly specific for PHD-mediated hydroxylation [17]. A significant body of findings has extended these initial evidences. So far other different proteins were described to interact with pVHL upon hydroxylation of specific proline residues [18–20,22], which in turn are targets of PHD enzymes (figure 2). It is almost clear that different linear motifs mediate the interaction with pVHL and collectively can be referred as hydroxy-degrons. The hydroxy-degron, similarly to what observed for other post-translational modification (PTM)-mediated degrons (e.g. the phospho-degron) requires direct activation by PTM, in this case promoted by 4-prolyl-hydroxylase enzymes. Consistent with the evidence that hypoxia conditions dictate the arrest of HIF-1*α* degradation, it can be argued that a range of diverse oxygen concentrations within different tissues may differentially impair hydroxy-degrons function. Because rapidly growing cells in developing organs and tumours experience hypoxia are easy to understand how hydroxy-degrons impairment may affect cancer progression.

#### 1.4.1. Protein sprouty homologue 2

Patel and co-workers demonstrated that protein sprouty homologue 2 (Spry2) is under PHD/pVHL-mediated regulation [20]. Spry2 is a modulator of MAPK/ERK pathway [87] acting as growth factors antagonist [88]. It mediates the endothelial quiescence and barrier integrity in endothelial cells and is suspected to play a role in cancer progression. PHD hydroxylate Spry2 on three Pro residues (i.e. 18, 144 and 160) to form three putative hydroxy-degrons that share poor sequence similarity with the LxxLAP motif (figure 2). Unfortunately, the author did not investigate which region of pVHL mediates the interaction. Considering the dependency from proline hydroxylation it can be presumed that the mediator of pVHL/Spry2 interaction is the HIF-1*α* recognition surface. Intriguingly, Spry protein family is thought to inhibit activation of ERK in response to FGF [89] VEGF [90]. This suggests pVHL to perform a double regulation of VEGF. One promoting HIF-1α-dependent expression of VEGF, while on the other side, the Spry2 stabilization induced by hypoxic conditions may itself serves to co-modulate the VEGF action.

#### 1.4.2. Erythropoietin receptor

Recently, another example of hydroxy-degron was described by Ohh and co-workers for the erythropoietin receptor (EPOR) [22]. EPOR is hydroxylated on Pro419 and Pro426 via PHD3, the resulting degrons have no sequence identity to LxxLAP motif, while localize within a proline-rich region (figure 2). The functional importance of this novel interaction is given by detailed mutagenesis investigations. Indeed, the authors reported examples of pVHL mutants that retain proper binding and regulation of HIF-1*α* while showing severe defect in binding EPOR. In particular, pVHL mutations classified as promoting VHL disease sub-phenotype 2C, showed a marked defect in binding to hydroxylated EPOR [22]. These findings suggest at least one novel molecular framework to explain the wide phenotypic variability in VHL disease. EPOR is the receptor for the erythropoietin (EPO) hormone whose gene is transactivated by HIF-2*α* [91] and mediates erythropoietin-induced erythroblast proliferation and differentiation. EPOR binds EPO upon phosphorylation-dependent conformational change mediated by JAK2 (Janus Kinase 2) [92]. Mutations of EPOR are causative of familiar erythrocytosis [93,94], which is also a common manifestation of either VHL disease [95] and hypoxia-sensing impairment [96–98]. Indeed, mutations of pVHL are also known to promote congenital erythrocytosis (e.g. the Chuvash polycythemia [99,100], a familiar hypoxia-sensing disorder characterized by increased production of red cells). It was proposed that pVHL forms with SOCS1 a heterodimeric E3 ligase complex which targets JAK2 for degradation [101]. The same authors also report that two pVHL homozygous mutations (i.e. p.Arg200Trp and p.His191Asp) are causative of erythrocytosis by impairing pVHL-SOCS1 association and yielding JAK2 stabilization [101]. Collectively, these data point to the existence of a signalling axis formed by VHL-EPOR/JAK/SOCS/STAT3 which may play important roles in supporting cancer cells proliferation. In this direction, it is interesting that recent work from Bento *et al.* where pVHL was demonstrated to also regulate JAK2/STAT3 signalling pathway in hemangioblastoma cells [102].

#### 1.4.3. Beta2-adrenergic receptor

Another hydroxy-degron is associated with hypoxia-regulated beta2-adrenergic receptor (ADRB2) [18] degradation. Beta-adrenergic receptors (*β*AR) play a relevant role in the regulation of cardiovascular and lung function, and *β*AR impairment is associated with heart failure [103] and pulmonary diseases [104]. ADRB2 is also thought to modulate the intracellular oxygen homeostasis modulating the AMP/ATP ratio, ROS production and PHD activity [105,106]. PHD3 hydroxylates ADRB2 in position Pro382 and Pro395 yielding formation of two pVHL binding sites. These, similarly to the other hydroxy-degron discussed here present low similarity with the LxxLAP motif of HIF-1*α* reinforcing the observation that pVHL β-domain can be evolved to recognize different hydroxy-motifs. It has been proposed that ADRB2 is critical for the modulation of hypoxia response as beta blocker treatment significantly reduces HIF-1*α*–specific binding to promoter sequences [106]. Due to its role in oxygen sensing, the PHD3–ADRB2–pVHL axis might be operating in cancer progression and deserves deeper investigation.

#### 1.4.4. Myb-binding protein 1A

The Myb-binding protein 1A (MYBBP1A) is a transcriptional regulator that interacts with DNA-binding proteins; in particular it regulates AhR-dependent gene expression [107], suppresses mitochondrial respiration [108] and in concert with CRY1 acts as a co-repressor of the Period2 promoter in mammals [109]. Hydroxylation of Pro695 of MYBBP1A activates a degron signal which promotes its pVHL-mediated degradation [19]. The role of MYBBP1A in cancer insurgence is not completely clear; however, recent studies linked this nuclear protein to the regulation of anoikis [110], a particular programmed cell death that occurs when specific cell detach from the surrounding extracellular matrix (ECM). pVHL is known to regulate ECM deposition [111] and defect in the hypoxia-depend regulation of MYBBP1A may have a role in cancer progression.

## 2. Concluding remarks and future perspectives

In conclusion, the emerging notion from the studies highlighted here is that the pVHL can mediate different cell functions depending on the biochemical context in which is intervening. pVHL, as an E3 component, seems to preferentially interact with hydroxylated proteins, a behaviour that suggests the existence of an entire novel class of hydroxylation-dependent degrons. Shedding light on these alternative degradation targets should help in understanding the role of pVHL on cancer progression as well as suggesting new therapeutic approaches for cancer treatment. A recent example of how this knowledge can be used is given by the application of the PROTAC (proteolysis-targeting chimeras) technique in development of a mimetic synthetic compound, which uses hydroxyl-proline to induce pVHL-mediated degradation of protein targets [112]. Although promising, these approaches should stimulate some reflection on the use of such compounds (e.g. VEGF antagonist, hypoxia inducer) when not supported by an exhaustive knowledge of all pathways involved. Further, the body of knowledge presented in this review, although extending pVHL role in degrading different targets, does not support the implication of having relevant fractions of free pVHL during hypoxic conditions. An alternative pVHL role as kinase inhibitor is suggested in both normoxia and hypoxia by several studies. However, this is a still open question that should be addressed in the next future. These are questions that will probably keep pVHL researchers occupied for the next few years.

## Data accessibility

This article has no additional data.
